# Sirt1-inducible deacetylation of p21 promotes cardiomyocyte proliferation

**DOI:** 10.18632/aging.102587

**Published:** 2019-12-26

**Authors:** Bing Li, Mengsha Li, Xinzhong Li, Hairui Li, Yanxian Lai, Senlin Huang, Xiang He, Xiaoyun Si, Hao Zheng, Wangjun Liao, Yulin Liao, Jianping Bin

**Affiliations:** 1 Department of Cardiology, State Key Laboratory of Organ Failure Research, Nanfang Hospital, Southern Medical University, Guangzhou 510515, China; 2 School of Medicine, Guizhou University, Guiyang, Guizhou 550025, China; 3 Guangzhou Regenerative Medicine and Health Guangdong Laboratory, Guangzhou 510005, China; 4 Department of Oncology, Nanfang Hospital, Southern Medical University, Guangzhou 510515, China

**Keywords:** cardiomyocyte proliferation, cardiac regeneration, myocardial infarction, Sirt1, p21

## Abstract

Inducing cardiomyocyte proliferation is a hopeful approach for cardiac regeneration following myocardial infarction. Previous studies have shown that p21 inhibits the cardiomyocyte proliferation and cardiac regeneration. Deacetylation of p21 by Sirt1 deacetylase may reduce p21 abundance and remove p21-induced cell cycle arrest. However, whether p21 deacetylation and Sirt1 deacetylate control cardiomyocyte proliferation is unclear. Here, we show that acetylation of p21 induces cardiomyocyte proliferation arrest, whereas blocking the acetylation of p21 increases cardiomyocyte proliferation. P21 can be acetylated by Sirt1, and Sirt1 activate p21 ubiquitination through deacetylation. Additionally, overexpression of Sirt1 induces EdU-, pH3-, and Aurora B-positive cardiomyocytes in neonatal and adult mice. In contrast, depletion of Sirt1 reduces cardiomyocyte proliferation *in vitro* and *in vivo*. Moreover, Sirt1 protects cardiac function, reduces cardiac remodeling, inhibits cardiomyocyte apoptosis, and attenuates cardiomyocyte hypertrophy post-myocardial infarction. These results suggest that Sirt1-induced p21 deacetylation plays an essential role in cardiomyocyte proliferation and that it could be a novel therapeutic strategy for myocardial infarction.

## INTRODUCTION

Acute myocardial infarction (MI) causes the rapid death of cardiomyocytes (CMs) with subsequent cardiac dysfunction. Approaches that could induce cardiac regeneration would help increase the number of CMs for the recovery of cardiac function post-MI [1–3]. Indeed, recent studies have demonstrated that the induction of CM proliferation could be a promising way for inducing cardiac regeneration [4, 5].

P21, encoded by cyclin-dependent kinase inhibitor 1A, directly inhibits G1, S, and G2 phase cyclin-dependent kinases in the cell cycle [6–8]. Previous studies have revealed that p21 inhibits various types of cancer cell cycles [9] and skeletal muscle [10], and bone regeneration [11]. P21 also exhibits a regeneration-inhibiting role in mammalian heart [12, 13]. These reports indicate that p21 plays a powerful inhibitory role in tissue proliferation and regeneration. A previous study showed that p21 can be acetylated by Tip60 in HCT116 cells [14]. The acetylation of proteins is known to occur on lysine residues, which are targets for ubiquitination and degradation [15]. Hence, acetylation makes p21 more stable and unable to be degraded by ubiquitination followed by HCT116 cell cycle arrest [14]. When acetylation proteins become deacetylated, proteins could be depleted by ubiquitination [15]. Herein, the deacetylation of p21 may lead to p21 degradation, reducing p21 abundance, removing CM proliferation arrest, and ultimately promoting CM proliferation. However, the deacetylation of p21 in the CM proliferation has not been studied.

Sirtuins (Sirt1-7) are a family of histone deacetylases (HDACs) that catalyze the deacetylation of protein lysine residues [16]. Sirtuins are involved in many vital processes of cells including tissue regeneration [17], cell cycle regulation [18], DNA repair [19], and tumorigenesis [20]. Not only is Sirtuin1 (Sirt1) crucial for cardiac embryogenesis and development [21, 22], but it also contributes to the cell proliferation during deacetylating targets [23] in previous studies. The importance of Sirt1 during cardiac development is highlighted by the cardiac defects associated with the perinatal death of Sirt1-deficient mice that rarely survive postnatally and exhibit developmental abnormalities in the heart [22]. Studies on cancer have revealed that Sirt1 triggers the cell proliferation through deacetylating target proteins such as p53 and Foxo1, thereby regulating tumor cell proliferation and tumorigenesis [23, 24]. In addition, Sirt1 deacetylates FOXO family members [25], p53 [26], and eIF2α [27], which regulate cell survival and cellular stress response in CMs. The interaction of HDACs with p21 was reported in a recent study, suggesting a potential deacetylation process for p21 [28]. Therefore, Sirt1 may induce the CM proliferation by deacetylating p21 and thereby cardiac regeneration.

In this study, we hypothesized that Sirt1 could induce CM proliferation and cardiac regeneration through deacetylating p21. The purpose of our study was to elucidate whether the deacetylation of p21 can attenuate CM proliferation arrest, and whether Sirt1 can deacetylate and ubiquitinate p21, thereby inducing CM proliferation and cardiac regeneration. Our study provides a novel strategy which could be used as an effective therapeutic approach following MI.

## RESULTS

### P21 is acetylated in CMs and p21 deacetylation attenuates CM proliferation arrest

**Figure 1:**
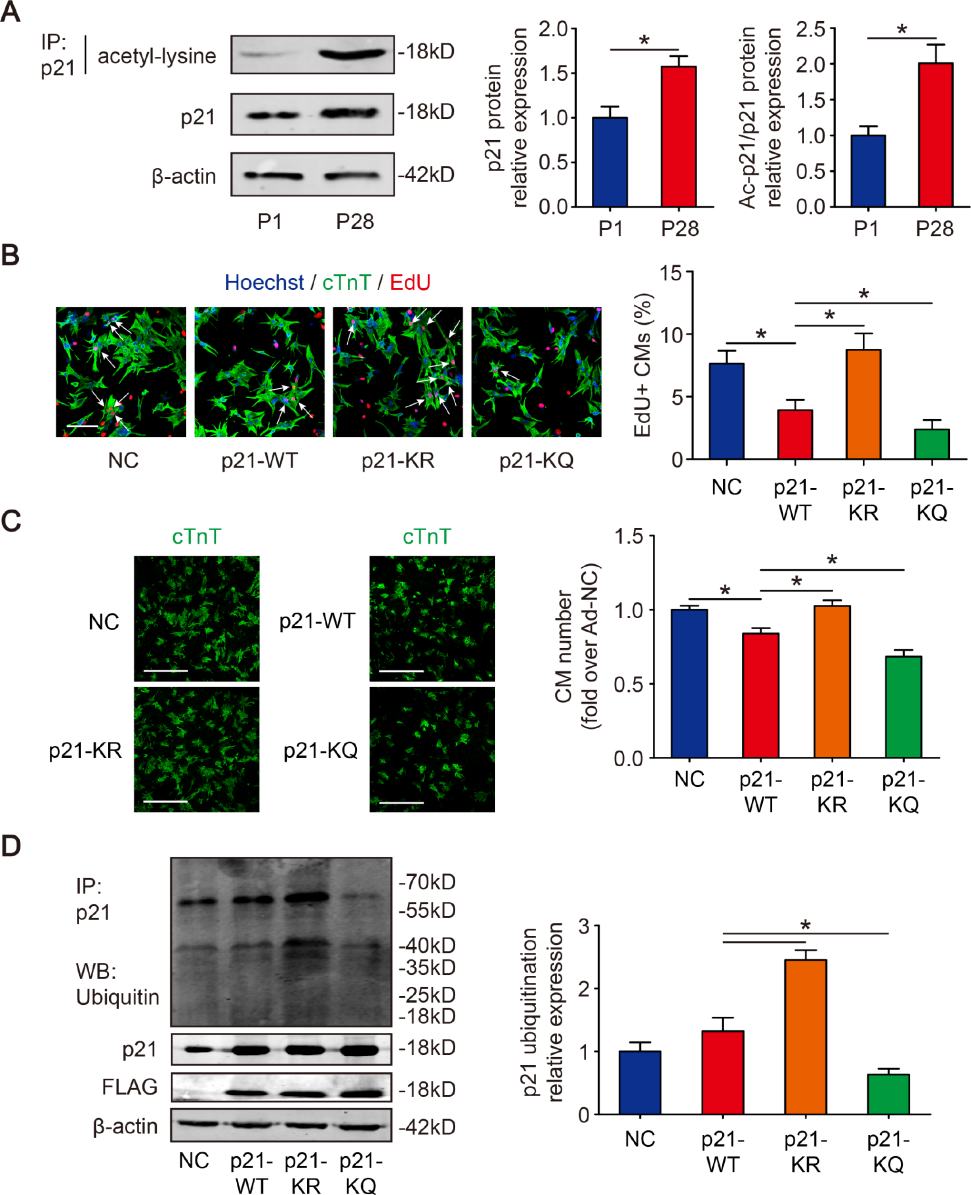
**Deacetylation of p21 attenuates p21 stability and CM cell cycle arrest.** (**A**) P1 and P28 mouse heart lysates were immunoprecipitated with a p21 antibody and analyzed by Western blotting with acetyl-lysine antibody; Western blotting was used to evaluate p21 protein expression in P1 and P28 mouse heart lysates. β-actin was used as a loading control (n=5). (**B**) Isolated P1 CMs were transfected with NC, p21-WT, p21-KR, or p21-KQ. EdU incorporation was detected by immunofluorescence. Scale bar, 50 μm. Quantitative analyses represent fields from 5 mice per group. (**C**) Isolated P1 CMs were transfected with NC, p21-WT, p21-KR, or p21-KQ and quantification of counts from P1 CMs transfected with NC, p21-WT, p21-KR, or p21-KQ (n=5). cTnT immunofluorescence was assessed to detect CM numbers. Scale bar, 500 μm. (**D**) Isolated P1 CMs were transfected with NC, FLAG-p21-WT, FLAG-p21-KR or FLAG-p21-KQ. Whole cell lysates were immunoprecipitated with a p21 antibody and analyzed by Western blotting with an ubiquitin antibodies; Western blotting was used to detect p21 levels. β-actin was used as a loading control (n=5). Statistical significance was calculated using a two-tailed unpaired Student’s t-test in (**A**) and a one-way ANOVA followed by an LSD post hoc test in (**B**–**D**). *p<0.05; data are presented as the mean ± S.E.M.

We first examined the acetylation level of p21 in the hearts of mice of different ages. P21 was pulled down from mouse heart lysates Post-natal day 1 (P1) and post-natal day 28 (P28); then, the proteins acetylated on lysine residues were detected. The immunoprecipitate results showed that the acetylation of p21 in P28 CMs was 2.01±0.26-fold higher than in P1 CMs (Figure 1A). The Western blotting results showed that the p21 level in P28 CMs was 1.57±0.12-fold higher than in P1 CMs (Figure 1A). To identify p21 acetylation sites, the immunoprecipitate gels from Figure 1A were analyzed by nanoLC-MS/MS. The analysis revealed that p21 has two potential acetylation sites, namely, the lysine residues positioned at sites 156 and 158 (Supplementary Figure 1A). Moreover, the two lysine sites are conserved across various mammalian species including mouse, rat and human (Supplementary Figure 1B). We further examined whether acetylation/deacetylation has an impact on CM proliferation. Isolated P1 CMs were transfected with FLAG-p21-WT or deacetylation-mimic FLAG-p21-KR or acetylation-mimic FLAG-p21-KQ, and then DNA synthesis was detected by 5-ethynyl-2’-deoxyuridine (EdU) incorporation. We found that FLAG-p21-WT decreased EdU-positive CMs, whereas FLAG-p21-KR showed a significance increase in the percentage of EdU compared with FLAG-p21-WT, and FLAG-p21-KQ decreased the EdU-positive CMs compared with FLAG-p21-WT (Figure 1B). Next, we quantified CM numbers and found that FLAG-p21-WT inhibited the raise of CM number, FLAG-p21-KR increase the CM number and FLAG-p21-KQ decrease the CM number compared with FLAG-p21-WT in the day 6 post-isolation (Figure 1C). We further examined whether p21 acetylation/deacetylation can participate in the regulation of p21 ubiquitination. Isolated P1 CMs were transfected with FLAG-p21-WT, FLAG-p21-KR or FLAG-p21-KQ. The immunoprecipitation results revealed that FLAG-p21-KR showed higher levels of ubiquitination compared to FLAG-p21-WT, whereas FLAG-p21-KQ showed low levels of ubiquitination compared to FLAG-p21-WT (Figure 1D). These results suggested that the deacetylation of p21 removes p21-induced CM proliferation arrest.

### Sirt1 interacts with and deacetylates p21

**Figure 2:**
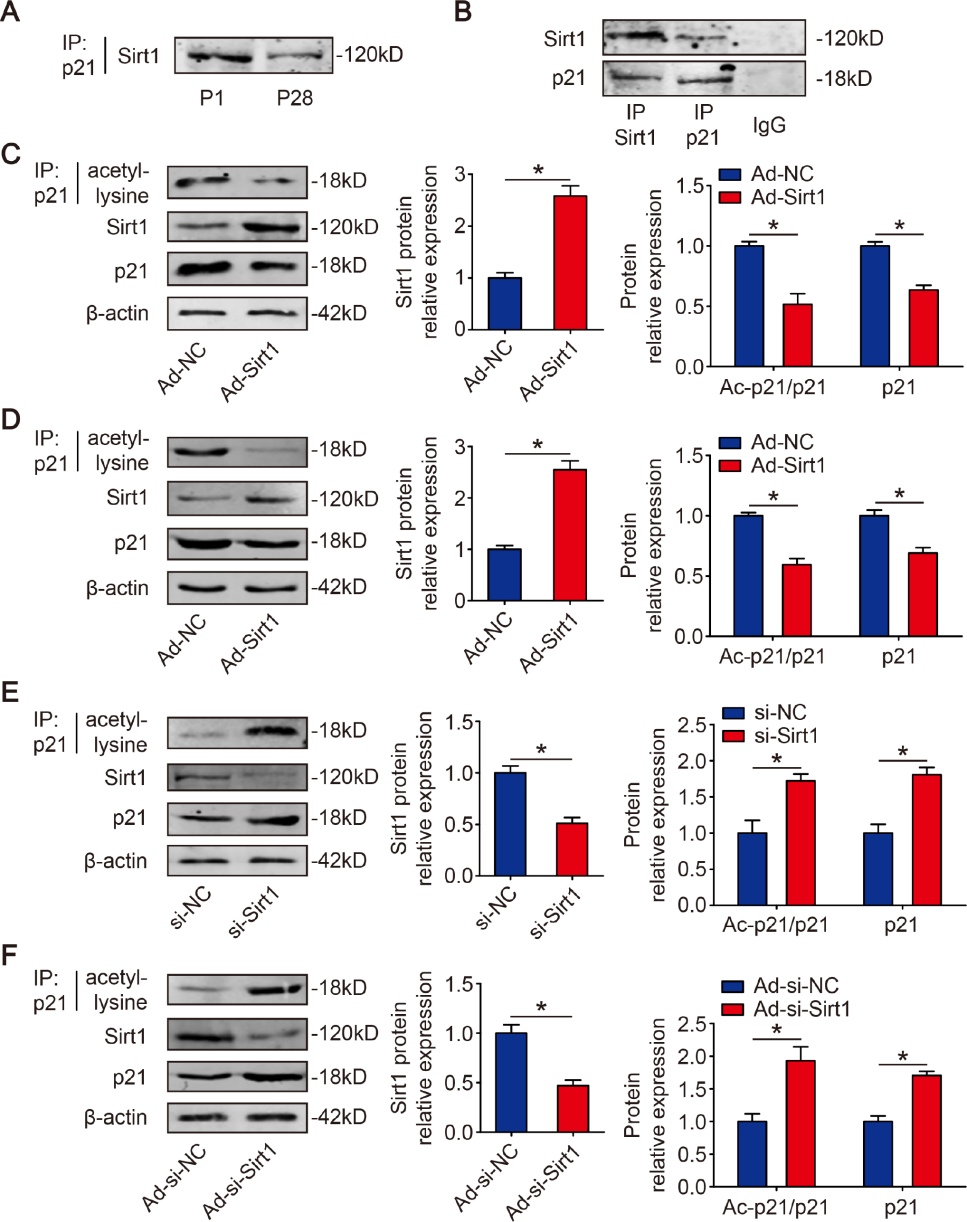
**p21 is deacetylated by Sirt1 in CMs.** (**A**) P1 and P28 mouse heart lysates were immunoprecipitated with a p21 antibody and analyzed by Western blotting with Sirt1 antibody (n=5). (**B**) Sirt1 was precipitated from isolated P1 CMs with Sirt1 antibody and blotted with p21 antibody, and vice versa (n=5). Negative controls: IgG. (**C**) Isolated P1 CMs were transfected with Ad-NC or Ad-Sirt1. Whole cell lysates were immunoprecipitated with a p21 antibody and analyzed by Western blotting using an acetyl-lysine antibody; Western blotting was performed to assess Sirt1 and p21 protein expression in Ad-NC or Ad-Sirt1 P1 CMs. β-actin was used as a loading control (n=5). (**D**) Adult mouse hearts were injected with Ad-NC or Ad-Sirt1. Heart lysates were immunoprecipitated with a p21 antibody and analyzed by Western blotting with an acetyl-lysine antibody; Western blotting was performed to evaluate Sirt1 and p21 protein expression in Ad-NC or Ad-Sirt1 adult heart lysates (n=5). (**E**) Isolated P1 CMs were transfected with si-NC or si-Sirt1. Whole cell lysates were immunoprecipitated with a p21 antibody and analyzed by Western blotting using an acetyl-lysine antibody; Western blotting was performed to evaluate Sirt1 and p21 protein expression in si-NC or si-Sirt1 P1 CMs (n=5). (**F**) P1 mouse hearts were injected with Ad-si-NC or Ad-si-Sirt1. Heart lysates were immunoprecipitated with a p21 antibody and analyzed by Western blotting using an acetyl-lysine antibody; Western blotting was performed to evaluate Sirt1 and p21 protein expression in Ad-si-NC or Ad-si-Sirt1 adult heart lysates (n=5). Statistical significance was calculated using a two-tailed unpaired Student’s t-test in **C**–**F**. *p<0.05; data are presented as the mean ± S.E.M.

Sirt1 is known to regulate protein activity through deacetylation on lysine residues [29]. Next, we determined whether Sirt1 and p21 physically interact with each other in CMs, co-immunoprecipitation experiments were carried out. We found that Sirt1 can clearly be co-immunoprecipitated by p21 in mouse hearts (Figure 2A). Immunoprecipitation of endogenous Sirt1 from isolated P1 mouse CMs co-precipitated p21 (Figure 2B). The reverse experiment, immunoprecipitation endogenous p21 and immunoblotting for Sirt1, confirmed their directly interaction (Figure 2B). Then, we determined whether Sirt1 deacetylates p21 in CMs. We overexpressed Sirt1 in isolated P1 CMs using adenovirus vectors. The Western blotting results showed that Ad-Sirt1 significantly overexpressed the Sirt1 protein level to 2.58±0.20-fold in P1 CMs (Figure 2C). The RT-qPCR results revealed that Ad-Sirt1 decreased p21 mRNA level in P1 CMs (Supplementary Figure 3A). The immunoprecipitation results showed that Ad-Sirt1 decreased the acetylation level of p21 to 51.6±8.8% in P1 CMs, and Ad-Sirt1 decreased total p21 expression to 63.5±3.9% (Figure 2C). In addition, Ad-Sirt1 decreased the quantity of acetylated peptides on K156 and K158 residues (Supplementary Figure 3B). Next, Ad-Sirt1 or Ad-negative control (Ad-NC) was injected into adult mouse hearts. At 10 days after injection, the Western blotting results revealed that Sirt1 was overexpressed to 2.70±0.27-fold in the Ad-Sirt1 hearts compared with the Ad-NC hearts (Figure 2D). The immunoprecipitation results showed that the acetylation level of p21 was depleted to 59.8±4.0% in adult Ad-Sirt1 hearts, and Ad-Sirt1 decreased the total p21 level to 68.3±4.6% in adult hearts (Figure 2D). We further knocked down Sirt1 using synthetic small interfering RNA (siRNA) in P1 CMs *in vitro*. In order to test the efficiency and specificity of synthetic Sirt1 siRNAs to silence endogenous Sirt1, two siRNAs were initially designed for Sirt1. We transfected each of the two different Sirt1 siRNAs and mismatched control siRNA, respectively, into isolated P1 CMs, and found that si-Sirt1-1 can silence Sirt1 mRNA and protein expression effectively and specifically (Supplementary Figure 2A, 2B). Therefore, we chose the effectual si-Sirt1-1 for subsequent si-Sirt1 experiments. The RT-qPCR results revealed that si-Sirt1 increased p21 mRNA level in P1 CMs (Supplementary Figure 3C). The acetylation of p21 and total p21 was significantly increased (1.72±0.09-fold, 1.81±0.10-fold, respectively) when P1 CMs were depleted with Sirt1 (Figure 2E). Further, si-Sirt1 increased the quantity of acetylated peptides on K156 and K158 residues (Supplementary Figure 3D). In addition, the knockdown of Sirt1 using shRNA adenovirus in P1 mice (Figure 2F) produced similar results. At 10 days after the injection of adenovirus, the depletion of Sirt1 showed a significant increase in the acetylation of p21 and total p21 (Figure 2F). These results indicated that p21 is deacetylated by Sirt1 in CMs.

### Sirt1 promotes p21 ubiquitination

**Figure 3:**
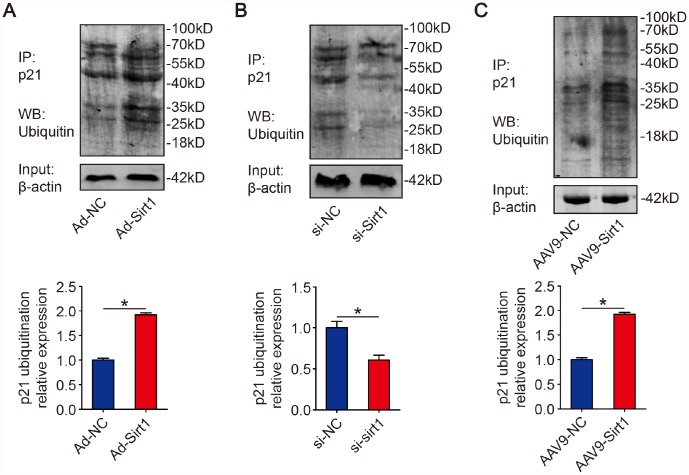
**Sirt1 promotes p21 ubiquitination.** (**A**) Isolated P1 CMs were transfected with Ad-NC or Ad-Sirt1. Whole cell lysates were immunoprecipitated with a p21 antibody and analyzed by Western blotting using an ubiquitin antibody (n=3). (**B**) Isolated P1 CMs were transfected with si-NC or si-Sirt1. Whole cell lysates were immunoprecipitated with a p21 antibody and analyzed by Western blotting using an ubiquitin antibody (n=3). (**C**) 8-week-old mouse hearts were injected with AAV9-NC or AAV9-Sirt1. Heart lysates were immunoprecipitated with a p21 antibody and analyzed by Western blotting using an ubiquitin antibody (n=5). Statistical significance was calculated using two-tailed unpaired Student’s t-test in (**A**–**C**). *p<0.05; data are presented as the mean ± S.E.M.

Next, we examined whether Sirt1 is involved in p21 ubiquitination. Isolated CMs were transfected with Ad-NC or Ad-Sirt1. We found that overexpression of Sirt1 increased ubiquitination of p21 (Figure 3A). On the contrary, p21 ubiquitination level was decreased when Sirt1 was depleted in isolated CMs (Figure 3B). Moreover, in adult mouse hearts, we found that adeno-associated virus serotype 9-Sirt1 (AAV9-Sirt1) increased the ubiquitination levels of p21 (Figure 3C). These data suggested that Sirt1 activate p21 ubiquitination.

### Sirt1 promotes neonatal CM proliferation

**Figure 4:**
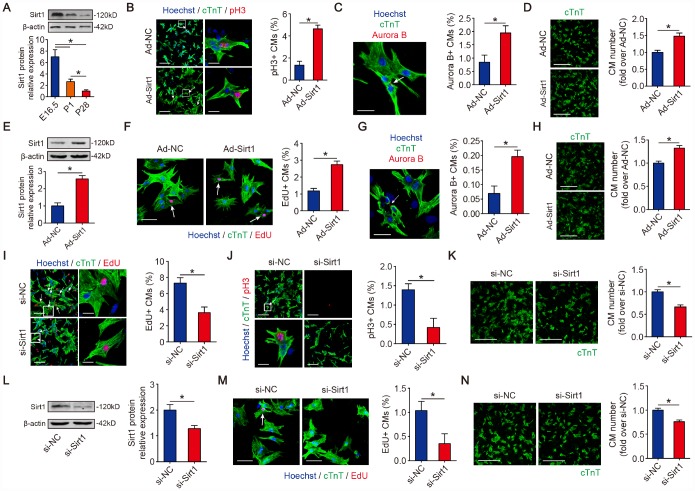
**Sirt1 induces CM proliferation *in vitro*.** (**A**) Western blotting and quantitative analyses of Sirt1 levels in E16.5, P1 and P28 mouse hearts. β-actin was used as a loading control (n=5). (**B**) Immunofluorescence of pH3 in P1 CMs transfected with Ad-NC or Ad-Sirt1 and quantification of pH3-positive CMs. pH3-positive CMs are indicated by arrows. Scale bar, left, 100 μm, right, 20 μm. Quantitative analyses are representative of fields from 5 mice per group. (**C**) Immunofluorescence of Aurora B in P1 CMs transfected with Ad-NC or Ad-Sirt1 and Aurora B-positive CMs were quantified. The arrow indicates a CM cell division, in which Aurora B localizes at the midbody during cytokinesis. Scale bar, 20 μm. Quantitative analyses are representative of fields from 5 mice per group. (**D**) Immunofluorescence was performed for cTnT and quantification of the counts of P1 CMs transfected with Ad-NC or Ad-Sirt1 (n=5), Scale bar, 500 μm. (**E**) Western blotting and quantitative analyses of Sirt1 levels in P6 CMs transfected with Ad-NC or Ad-Sirt1. β-actin was used as a loading control (n=5). (**F**) Immunofluorescence for EdU in P6 CMs transfected with Ad-NC or Ad-Sirt1. EdU-positive CMs were quantified. Scale bar, 50 μm. EdU-positive CMs are indicated with arrows. Quantitative analyses are representative of fields from 5 mice per group. (**G**) Immunofluorescence for Aurora B in P6 CMs transfected with Ad-NC or Ad-Sirt1. Aurora B-positive CMs were quantified. The arrow indicates a CM cell division, in which Aurora B localizes at the midbody in cytokinesis. Scale bar, 50 μm. Quantitative analyses are representative of fields from 5 mice per group. (**H**) Immunofluorescence for cTnT and quantification of the counts of P6 CMs transfected with Ad-NC or Ad-Sirt1 (n=5), Scale bar, 500 μm. (**I**) DNA synthesis was assessed using EdU immunofluorescence staining and EdU-positive CMs were quantified in Sirt1-silenced P1 CMs. Scale bar, up, 100 μm, down, 20μm. Quantitative analyses are representative of fields from 5 mice per group. (**J**) Mitosis was detected using pH3 immunofluorescence staining and pH3-positive CMs were quantified in Sirt1-silenced P1 CMs. pH3-positive CMs are indicated by arrows. Scale bar, up, 100 μm, down, 20μm. Quantitative analyses are representative of multiple fields from 5 mice per group. (**K**) Immunofluorescence for cTnT and quantification of the counts of P1 CMs transfected with si-NC or si-Sirt1 (n=5), Scale bar, 500 μm. (**L**) Western blotting and quantitative analyses of Sirt1 levels in P6 CMs transfected with si-NC or si-Sirt1. β-actin was used as a loading control (n=5). (**M**) Immunofluorescence for EdU and quantification of EdU-positive CMs in P6 CMs transfected with si-NC or si-Sirt1. The arrow indicates EdU-positive CM nuclei. Scale bar, 50 μm. Quantitative analyses are representative of fields from 5 mice per group. (**N**) Immunofluorescence for cTnT and quantification of the counts of P6 CMs transfected with si-NC or si-Sirt1 (n=5), Scale bar, 500 μm. Statistical significance was calculated using a one-way ANOVA followed by the LSD post hoc test in A and two-tailed unpaired Student’s t-test in B-N. *p<0.05; data are presented as the mean ± S.E.M.

Given that Sirt1 can deacetylate p21 in CMs, and p21 is considered a cell cycle inhibitor in CM proliferation, we hypothesize that Sirt1 is associated with CM proliferation. Publicly available databases (GSE50704, GSE51483, and GSE93269) show that Sirt1 is upregulated in mouse fetal hearts compared with adult hearts (Supplementary Figure 4A–4C). Then, we detected the expression pattern of Sirt1 in fetal, neonatal and adult mouse hearts. The real-time PCR and Western blotting results for mice revealed a high-level expression of Sirt1 in embryonic day 16.5 CMs and a sharp reduction in P1 CMs which was further reduced in P28 CMs (Figure 4A). Then, we detected whether the overexpression of Sirt1 can impact CMs proliferation *in vitro*. We used Ad-Sirt1 to overexpress Sirt1 in the following overexpression experiments *in vitro* (Figure 2C). We found an induction of CM proliferation as quantified by an increase in mitosis marker histone H3 phosphorylated at serine 10 (pH3)-positive CMs in the P1 CMs with overexpressed Sirt1 (4.64±0.33%) (Figure 4B). In addition, we found that the cytokinesis marker Aurora B kinase was markedly expressed in the cleavage furrow between proliferating CMs in the P1 CMs with overexpressed Sirt1 (1.95±0.27%) (Figure 4C). P1 CM numbers increased by 1.48±0.09-fold upon overexpression of Sirt1 (Figure 4D). Next, we assessed the effect of Sirt1 in Post-natal day 6 (P6) CMs. Sirt1 was overexpressed by 2.57±0.20-fold in P6 CMs transfected with Ad-Sirt1 compared to Ad-NC (Figure 4E). Compared to Ad-NC P6 CMs, EdU- and Aurora B-positive P6 CMs were significantly increased (1.18±0.29% to 2.73±0.43%, and 0.07±0.05% to 0.20±0.04%, respectively) in the Ad-Sirt1 group (Figure 4F, 4G). In addition, the Ad-Sirt1 group showed a 1.32±0.06-fold increase in P6 cell numbers (Figure 4H). We further examined the effect of siRNA mediated Sirt1 knockdown on CM proliferation. The depletion of Sirt1 (Figure 2E) significantly decreased EdU- and pH3-positive P1 CMs to 3.64±0.69% and 0.42±0.48%, respectively (Figure 4I, 4J). Moreover, si-Sirt1 reduced the P1 CM numbers to 66.3±4.6% of si-NC-treated P1 CM numbers (Figure 4K). Next, we depleted Sirt1 expression in P6 CMs using si-Sirt1 (Figure 4L). Sirt1 knockdown significantly decreased the percentage of EdU-positive P6 CMs to 0.35±0.41% (Figure 4M). Sirt1 depletion decreased cell numbers to 75.5±3.7% of the si-NC-treated P6 CMs (Figure 4N). These data showed that Sirt1 increases CM proliferation *in vitro*.

### Sirt1 induces CM proliferation *in vivo*

**Figure 5:**
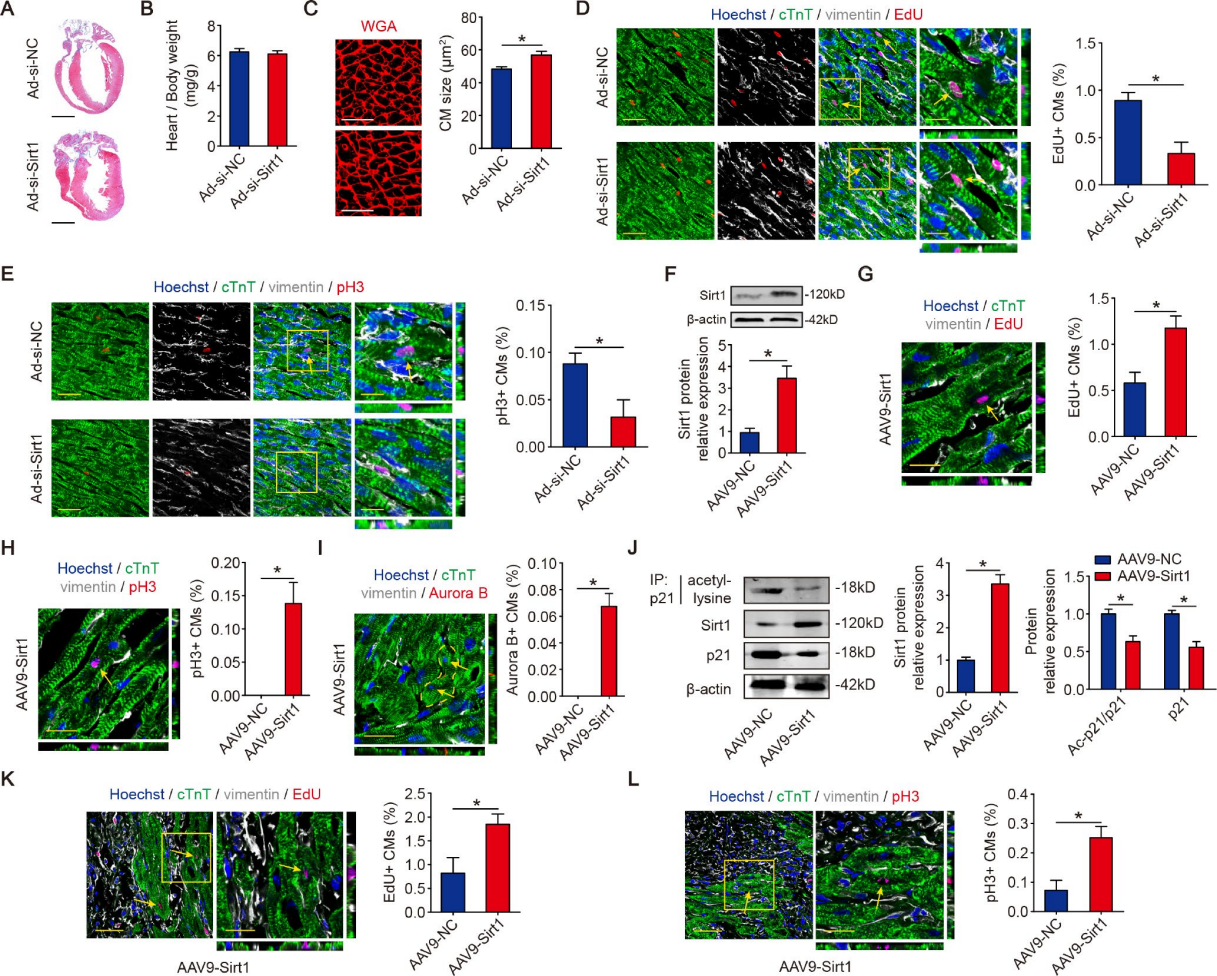
**Sirt1 drives CM proliferation *in vivo*.** (**A**) Masson staining of sagittal heart sections from neonatal mice injected with Ad-si-NC or Ad-si-Sirt1, Scale bar, 1mm. (**B**) Ratios of heart weight-to-body weight in neonatal mouse hearts injected with Ad-si-NC or Ad-si-Sirt1 (n=5). (**C**) WGA staining of sagittal heart sections in the Ad-si-NC and Ad-si-Sirt1 groups at P10. Quantitative analyses are representative of fields from 5 mice per group, Scale bar, 50μm. (**D**) Immunofluorescence for EdU and quantification of EdU-positive CMs in neonatal hearts injected with Ad-si-NC or Ad-si-Sirt1 (n=5). The arrows indicate EdU-positive CM nuclei. Scale bar, left, 50 μm, right, 20 μm. (**E**) Immunofluorescence for pH3 and quantification of pH3-positive CMs in neonatal hearts injected with Ad-si-NC or Ad-si-Sirt1 (n=5). The arrow indicates pH3-positive CM nuclei. Scale bar, left, 50 μm, right, 20 μm. (**F**) Western blotting analyses of Sirt1 levels in adeno-associated virus 9-negative control (AAV9-NC) or adeno-associated virus 9-Sirt1 (AAV9-Sirt1) injected adult mouse hearts 28 days after injection. β-actin was used as a loading control (n=5). (**G**) DNA synthesis was assessed using EdU immunofluorescence staining and EdU-positive CMs were quantified in AAV9-NC and AAV9-Sirt1 adult mouse hearts. The arrow indicates EdU-positive CM nuclei. Scale bar, 20μm. Quantitative analyses are representative of fields from 5 mice per group. (**H**) Mitosis was detected using pH3 immunofluorescence staining and pH3-positive CMs are quantified in AAV9-NC and AAV9-Sirt1 adult mouse hearts. The arrow indicates pH3-positive CM nuclei. Scale bar, 20μm. Quantitative analyses are representative of fields from 5 mice per group. (**I**) Cytokinesis was detected using Aurora B immunofluorescence staining and Aurora B-positive CMs were quantified in AAV9-NC and AAV9-Sirt1 adult mouse hearts. The dotted lines indicate the daughter cells and the arrows indicate their nuclei. Scale bar, 20μm. Quantitative analyses are representative of fields from 5 mice per group. (**J**) 8-week-old MI mouse hearts were injected with AAV9-NC or AAV9-Sirt1. Heart lysates were immunoprecipitated with a p21 antibody and analyzed by Western blotting using an acetyl-lysine antibody; Western blotting was performed to evaluate Sirt1 and p21 protein expression in AAV9-NC or AAV9-Sirt1 adult heart lysates (n=5). (**K**) DNA synthesis was assessed using EdU immunofluorescence staining and quantification of EdU-positive CMs in AAV9-NC and AAV9-Sirt1 adult mouse hearts 28 days after MI. The arrows indicate EdU-positive CM nuclei. Scale bar, 20μm. Quantitative analyses are representative of fields from 5 mice per group. (**L**) Mitosis was detected using pH3 immunofluorescence staining and pH3-positive CMs were quantified in AAV9-NC and AAV9-Sirt1 adult mouse hearts 28 days after MI. The arrows indicate pH3-positive CM nuclei. Scale bar, 20μm. Quantitative analyses are representative of multiple fields from 5 mice per group. Statistical significance was calculated using a two-tailed unpaired Student’s t-test in A-L. *p<0.05; data are presented as the mean ± S.E.M.

We next investigated the role of Sirt1 in neonatal mouse hearts *in vivo*. At 10 days after the injection of adenovirus, the depletion of Sirt1 (Figure 2F) showed no significance in the heart weight/body weight (Figure 5A, 5B). The CM size was increased in Sirt1 knockdown hearts (Figure 5C). The inhibition of Sirt1 levels also decreased cardiomyocyte cell cycle activity as detected by immunofluorescence for EdU incorporation and pH3 *in vivo* (Figure 5D, 5E). To investigate whether Sirt1 promotes CM proliferation in adult mice, we induced Sirt1 overexpression in adult mouse hearts by intracardiac-injected AAV9 vectors expressing Sirt1 (Figure 5F). The mouse hearts with Sirt1 overexpression were morphologically normal (Supplementary Figure 5A), and the heart weight/body weight was not significant in these mice (Supplementary Figure 5B). AAV9-Sirt1 increased the DNA synthesis marker EdU, mitosis marker pH3, and cytokinesis marker Aurora B in CMs to 1.17±0.26%, 0.14±0.07%, and 0.07±0.02%, respectively compared with AAV9-NC adult CMs (Figure 5G, 5I). To test whether Sirt1 is involved in the regulation of CM proliferation in response to ischemic injury, an MI model was established. We performed left anterior descending (LAD) permanent ligation on 8-week-old mice, and ST segment elevation was observed on electrocardiogram (ECG) when LAD was ligated and then injected, in the peri-infarcted area, with AAV9 vectors expressing Sirt1 or the control vector (Supplementary Figure 5C, 5D). The Western blotting results revealed that Sirt1 was upregulated in the MI hearts compared with the Sham group (Supplementary Figure 5E). The immunoprecipitation results showed that the acetylation level of p21 was lower in the MI hearts compared with the Sham hearts (Supplementary Figure 5E). Moreover, the CM size was bigger in the MI hearts compared with the Sham hearts (Supplementary Figure 5F). Next, we detected whether Sirt1 deacetylate p21 in adult MI mouse hearts. The immunoprecipitation results showed that AAV9-Sirt1 decreased the acetylation level of p21 and total p21 expression in adult MI mouse hearts (Figure 5J). Using an EdU incorporation assay, we found a marked increase in the EdU incorporation in the CMs of the border zone of hearts with Sirt1 overexpression (1.85±0.44% in AAV9-Sirt1 group. Figure 5K). A significant number of pH3-positive CMs was detected in the infarct border zone in hearts with Sirt1 overexpression (0.25±0.08% in AAV9-Sirt1 group. Figure 5L). These data showed that Sirt1 significantly increased CM proliferation *in vivo*.

### Overexpression of Sirt1 improves cardiac function in adult mouse hearts post-MI

**Figure 6:**
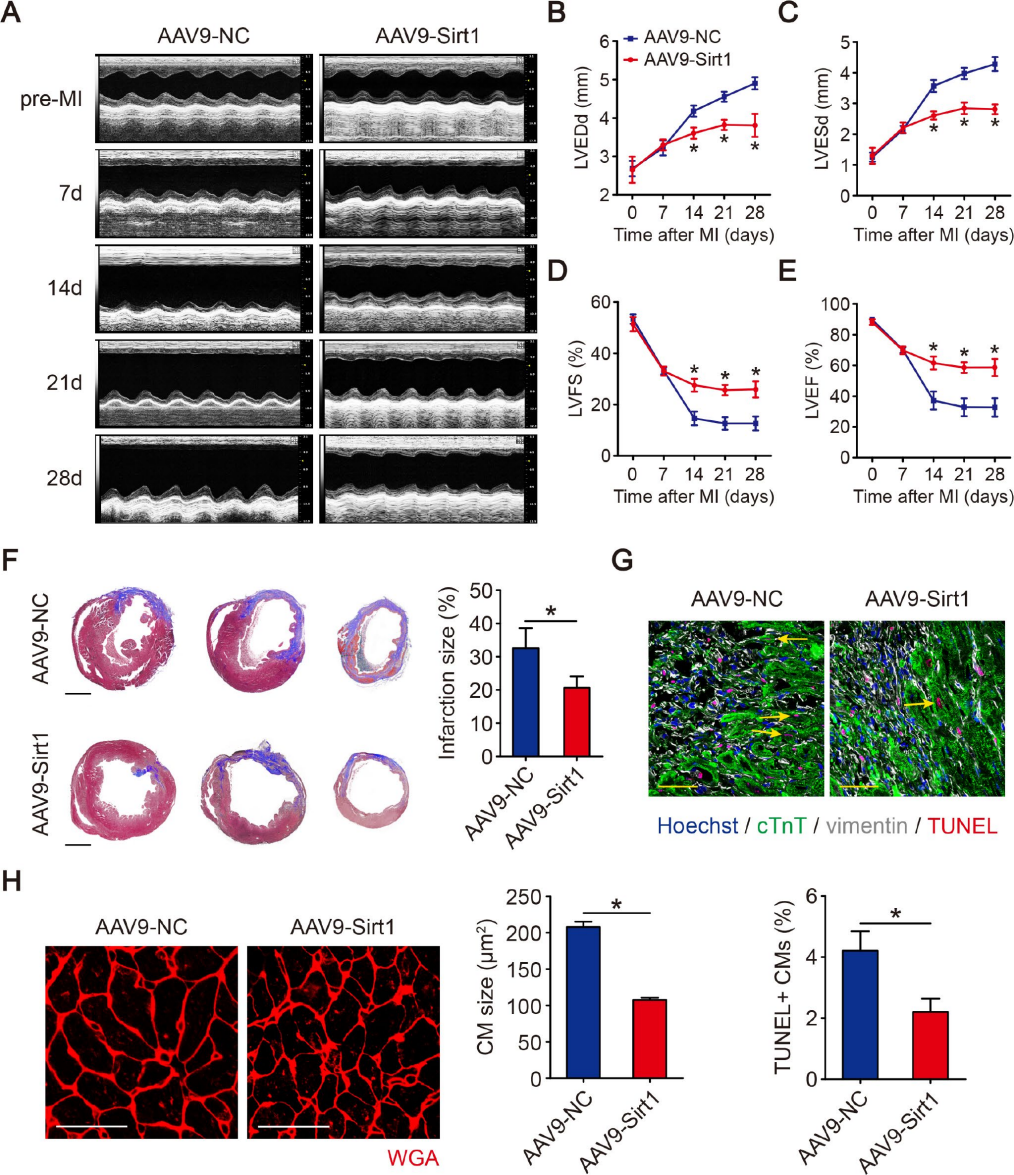
**Sirt1 induces cardiac regeneration after MI in adult mice.** (**A**–**E**) M-mode ultrasonic cardiography changes in mouse hearts, as detected by echocardiography in AAV9-NC and AAV9-Sirt1 adult mice pre-MI and 7, 14, 21, and 28 days after MI. Quantitative analyses were performed for LVEDd (**B**), LVESd (**C**), LVFS (**D**), and LVEF (**E**) (n=5). (**F**) Masson’s trichrome staining of serial ventricular sections in AAV9-NC and AAV9-Sirt1 adult mice 28 days after MI. Infarction size was quantified (n=5). Scale bar, 1mm. (**G**) Cell apoptosis was detected by TUNEL staining and TUNEL-positive CMs were quantified in AAV9-NC and AAV9-Sirt1 adult mice 28 days after MI. Scale bar, 20μm. Quantitative analyses are representative of fields from 5 mice per group. (**H**) WGA staining and quantitative analyses of CMs size in AAV9-NC and AAV9-Sirt1 adult mice 28 days after MI. Scale bar, 50 μm. Quantitative analyses are representative of fields from 5 mice per group. Statistical significance was calculated using a one-way ANOVA followed by LSD post hoc test in B-E and a two-tailed unpaired Student’s t-test in F-H. *p<0.05; data are presented as the mean ± S.E.M.

To evaluate whether Sirt1 improve left ventricular function in MI hearts, transthoracic two-dimensional echocardiography was performed on mice at 7, 14, 21 and 28 days after LAD infarction (Figure 6A, Supplementary Figure 5C). As evaluated by echocardiography, the ventricular chamber dilation, both in diastole and systole, was significantly less in hearts with Sirt1 overexpression (Figure 6B, 6C). The preserved chamber dimensions in hearts with Sirt1 overexpression were associated with significantly better left ventricular function, as reflected by preserved fractional shortening and ejection fraction (Figure 6D, 6E). The analysis of Masson’s trichrome staining clearly showed that the infarction size was significantly reduced in hearts with Sirt1 overexpression (32.6±6.0% in AAV9-NC group, 20.6±3.4% in AAV9-Sirt1 group, Figure 6F). The overexpression of Sirt1 inhibited CMs apoptosis after MI, as judged by TUNEL assays (Figure 6G). AAV9-Sirt1 also inhibited CM size post-MI (Figure 6H). The lung weight/body weight was significantly smaller in mice with Sirt1 overexpression than in control mice (Supplementary Figure 5G). These results suggested that upregulation of Sirt1 contributes to endogenous regeneration in adult hearts after ischemic injury.

## DISCUSSION

In the present study, we reported that Sirt1-induced p21 deacetylation acts as a novel role promoting CM proliferation. In detail, p21 can be regulated by deacetylation in post-translational modification, which removed CM proliferation arrest. Furthermore, Sirt1 decreases p21 expression during both the mRNA level in transcription and deacetylation/ubiquitination in post-translational modification. Additionally, Sirt1 is a positive regulator of cardiac regeneration post-MI. Overall, these findings indicate that p21 deacetylation by Sirt1 may be a novel, effective strategy for inducing endogenous cardiac regeneration and protecting the heart from ischemic heart disease.

P21 has been intensively studied in CM proliferation, and p21 exerts an inhibitory role in cardiac regeneration [12, 13]. Previous studies have revealed that genes such as Meis1 [5], Tbx20 [30], and REST [31] regulate the CM cell cycle focus on modulating the p21 mRNA level by transcriptional control, but the post-translational modification events of p21 are poorly understood. Our data provided a novel post-translational modification pattern on the p21 protein in CM proliferation. We found that p21 is hyper-acetylated in CMs, which suggests that CM cell cycle arrest may be associated with the acetylation of p21. More importantly, we demonstrate that inducing p21 acetylation decreases CM proliferation and cell cycle checkpoint cyclins. Contrarily, reducing p21 acetylation significantly increases CM proliferation and cell cycle checkpoint cyclins. These results suggest that CM cell cycle arrest in adults is activated by p21 acetylation, and deacetylation of p21 may be an important novel regulatory mechanism in the regulation of CM proliferation. The acetylation/deacetylation of proteins modulates a wide variety of cellular biological processes such as cell proliferation, cell survival and apoptosis [32]. The acetylation of proteins can block ubiquitination at the lysine residues to prevent proteasomal degradation there by making protein more stable, whereas the deacetylation of lysines may be a prerequisite for subsequent ubiquitination and degradation [33]. A direct relationship between the acetylation/deacetylation and ubiquitination of the same lysine residues has been reported for p53 [15], Runx3 [34], Smad7 [35], and so on. A study performed in HCT116 cells has shown that P21 acetylation by Tip60 counteracts the ubiquitination process of p21, enhancing p21 stability followed by cell cycle arrest [14]. In the present study, the acetylation of p21 may inhibit p21 degradation by the ubiquitin-proteasome pathway, exhibiting a high activity and abundance of p21 to retain function to inhibit CMs proliferation. On the contrary, the deacetylation of p21 may lead to degradation by the ubiquitin-proteasome pathway, which suppresses the p21 level and removes proliferation arrest in CMs. Collectively, our study revealed that p21 deacetylation participates in regulating CM proliferation, and p21 deacetylation could become a crucial direction for cardiac regeneration.

We further demonstrated that p21 is deacetylated by Sirt1 on lysine K156 and K158. Furthermore, Sirt1 induces CM proliferation by decreasing and deacetylating p21. Past studies have shown that Meis1 [5], Tbx20 [30], and REST [31] control p21 mRNA at the transcription level in regulating CM proliferation. In our study, Sirt1 can decrease p21 mRNA and the total p21 protein level in CMs. Consistent with our results, previous studies have also shown that Sirt1 can decrease the p21 level in skeletal muscle precursor cells [36], HCT116 cells [37], and so on, but no data on Sirt1 inducing p21 deacetylation have been reported. In the present study, we demonstrated that p21 is acetylated on lysine K156 and K158. To detect whether Sirt1 can directly deacetylates p21, co-immunoprecipitation assays was performed to detect protein-protein interactions. Our data showed that overexpression of Sirt1 decreased the acetylation of p21, while the depletion of Sirt1 increased p21 acetylation *in vitro* and *in vivo*. These results revealed that Sirt1 can directly interacts with and deacetylates p21. Overall, Sirt1 promotes CM proliferation drastically through reducing p21 not only at the transcription level but also at the post-translation level. A previous study has shown that early postnatal hearts expressed a higher level of Sirt1 deacetylase activity than did older hearts, suggesting that Sirt1 deacetylase activity is participated in the regulation of cardiac development [38]. Combined with previous studies and our data, Sirt1 could be an attractive deacetylase to induce p21 deacetylation/ubiquitination and depression in abundance.

Previous studies have shown that Sirt1 promotes cell proliferation in malignant glioma cells [39], melanoma cells [40], glial progenitor cells [41], primary porcine aortic endothelial [42], skeletal muscle precursor cells [36], endothelial progenitor cells [43], and so on. In our study, the *in vitro* and *in vivo* results clearly demonstrated that Sirt1 promotes CM proliferation and cardiac regeneration. We overexpressed Sirt1 at a low level (2.1-fold to 2.8-fold) *in vitro* and *in vivo*. Furthermore, we used adenovirus (AdV) vector injection for neonatal and AAV9 vector injection for adults *in vivo* to sustain the levels of Sirt1 for short-term and long-term expression, respectively. We examined CM proliferation using immunofluorescence. We used a diverse set of antibodies to distinguish between CMs and cardiac fibroblasts *in vivo*. We also visualized EdU-, pH3-, and Aurora B-positive CMs using Z-stack confocal images, which demonstrated that the signal of these markers indeed belong to the CMs. Our results reveal that overexpression of Sirt1 causes an increase in EdU-, pH3- and Aurora B-positive CMs, which are classical markers of DNA synthesis, mitosis and cytokinesis, respectively, used in previous studies [2, 5, 44]. On the contrary, the depletion of Sirt1 decreased EdU- and pH3-positive CMs. Moreover, our results show that Sirt1 overexpression non-CMs have no paracrine effect on CM proliferation. Thus, Sirt1 promotes CM proliferation likely as a result of primary CM proliferation and not due to paracrine action of non-CMs. Then, we detected the regenerative role of Sirt1 in MI mouse models. We used transthoracic two-dimensional echocardiography, Masson’s trichrome staining, TUNEL staining and wheat germ agglutinin (WGA) staining to evaluate left ventricular dysfunction, cardiac fibrosis, CM apoptosis and CM hypertrophy, respectively, post-MI. Our findings indicate that overexpression of Sirt1 improved cardiac function, reduced cardiac fibrosis, inhibited CM apoptosis, and depressed CM hypertrophy in adult mice post-MI. Consistent with our study, previous studies have demonstrated that Sirt1 can inhibit CM apoptosis [45] and protect the heart from ischemia/reperfusion [46]. Taken together, our data and previous studies forcefully demonstrated that Sirt1 promotes cardiac regeneration and attenuates cardiac remodeling post-MI, supporting our identification of Sirt1 as a novel molecule involved in cardiac regeneration.

The present study has several limitations. We performed the function of p21 acetylation/deacetylation in CM proliferation *in vitro*. It is better that when experiments were conducted on p21-/- knockout mice, and we will elucidate the effects between the acetylation/deacetylation of p21 and cardiac regeneration post-MI *in vivo* in further studies. Additionally, we demonstrated that p21 acetylation/deacetylation is involved in Sirt1-induced CM proliferation, whether p21 acetylation/deacetylation is involved in Sirt1-regulated CM hypertrophy and apoptosis remains unclear and requires further studies. In the present study, although we obtained that overexpression of Sirt1 is benefit for cardiac regeneration post MI, the accurate abundance of Sirt1 which promotes cardiac regeneration more effectively remains to be explored.

In summary, the present study demonstrates that p21 deacetylation by Sirt1 could attenuate proliferation arrest in CMs. In addition, overexpression of Sirt1 triggers CM proliferation and cardiac regeneration. We conclude that activating p21 deacetylation and ubiquitination by Sirt1 could be beneficial for treating MI.

## MATERIALS AND METHODS

### Ventricular CM isolation

All animal care and experimental procedures were approved by the Institutional Animal Care and Use Committee at Southern Medical University, and they followed the NIH Guidelines for the Care and Use of Laboratory Animals. Animal studies are reported in compliance with the ARRIVE guidelines [47, 48]. The mouse ventricular CM isolation was performed as previous study [2]. P1 and P6 C57BL/6J mice were purchased from the Laboratory Animal Center of Southern Medical University. P1 or P6 mice were anaesthetized by 2% isoflurane and cervical dislocation. Then, ventricles from neonatal mice were separated and cut into pieces and digested in 0.25% trypsin (Gibco, CA, USA) at 4°C overnight. Digestion was again performed in collagenase type II (Gibco, CA, USA) with BSA (Sigma, Darmstadt, Germany) in PBS at 37°C for 15 min twice under constant stirring, and the supernatant was collected after each step. The collected supernatant was centrifuged to separate the cells, which were re-suspended in DMEM/F12 1:1 medium (Hyclone, Utah, USA) supplemented with 10% FBS (Gibco, CA, USA) and 100 U/ml of penicillin (Sigma, Darmstadt, Germany) and 100 mg/ml of streptomycin (Sigma, Darmstadt, Germany). The collected cells were seeded onto 100 mm plastic dishes for 2 hour at 37°C in a humidified atmosphere of 5% CO2. The supernatant was then collected and re-suspended in DMEM/F12 media, counted and plated at the appropriate density.

### Plasmids and siRNA transfection

P21-WT, p21-KR (which was mutated the K156 and K158 to Arg 156 and Arg 158 residue) and p21-KQ (which was mutated the K156 and K158 to Gln 156 and Gln 158 residue) was cloned into pcDNA3.1-FLAG plasmids, which were generated by Vigene (Shandong, China). The sequences of each point mutation are as follows (mutated bases in lower case): p21-KR: CGCAGATTGGTCTTCTGCAgGAGAAgACCC; p21-KQ: CGCAGATTGGTCTTCTGC**c**AGAGA**c**AACCC. Si-NC, two Sirt1 siRNAs, and mismatched control siRNA (si-MM) were synthesized by Ribobio (Guangzhou, China). Isolated P1 or P6 mouse CMs were seeded at 70% confluence for immunofluorescence analysis and 40% confluence for CM number analysis. After 48h of culturing, 5μl Lipofectamine 2000 (Invitrogen, CA, USA) and 50 nM plasmids, or siRNAs were added to the Opti-MEM medium (Gibco BRL, Paisley, UK). The mixed solution was incubated at room temperature for 20min and then added to the cells. This medium was replaced after 6h of incubation at 37°C with the same volume of DMEM/F12 medium. After 48h, the cells were subjected to RNA or protein isolation, or immunofluorescence analysis.

### Injection of AdV or AAV9 vectors into neonatal or adult mice

The AdV-GFP overexpression or depletion Sirt1 and the AAV9 vector overexpression Sirt1 were synthesized by Vigene (Shandong, China). In the experiments using AdV vectors, neonatal C57BL/6J mice (post-natal day 1) were anesthetized by cooling on an ice bed for 5 min. After the fourth intercostal was dissected, mice were intracardiac injected with NC or Ad-si-Sirt1 at a dose of 2 × 10^10^ viral genome particles per animal using an insulin syringe with an incorporated 30-gauge needle (BD, NJ, USA). The virus was injected into the neonatal heart at 3 sites and the total volume injected into each heart was 20μl. Then, mice were placed under a heat lamp and warmed for several minutes until recovery. The hearts of the injected mice were collected 10 days after AdV injection. For long-distance experiments (up to 28 days), AAV9 vectors were used for transduction *in vivo*. Intracardiac injection of AAV9 vectors (AAV9-NC or AAV9-Sirt1 or AAV9-si-NC or AAV9-si-Sirt1) in 8-week-old (24-26 g) mice at a dose of 1 × 10^11^ viral genome particles per animal was performed as described below for the animals that underwent MI.

### Western blotting

Western blotting was performed as previously reported [49]. Isolated mouse CMs or dissected mouse ventricular heart tissue samples were lysed in ice-cold RIPA lysis buffer with protease inhibitors. Protein concentrations were determined with a BCA Protein Quantitative Analysis kit (Fudebio-tech, Hangzhou, China). Protein samples were separated by 8-12% SDS-PAGE and transferred onto polyvinylidene difluoride membranes (Millipore, USA). The membranes were incubated at room temperature for 2h in TBST containing 5% BSA. After blocking, the membranes were incubated with primary antibodies overnight at 4°C. The following primary antibodies were used: Sirt1 (Cell Signaling Technology, #3931, RRID: AB_1642293, MA, USA), p21 (Abcam, ab188224, RRID: AB_2734729, Cambridge, UK), FLAG (Cell Signaling Technology, #14793, RRID: AB_ 2572291, MA, USA) and β-actin (Biosynthesis, bs-0061R, RRID: AB_10855480, Beijing, China). Then the membranes were washed three times with TBST, they were incubated with a donkey anti-rabbit IgG H&L antibody (Abcam, ab16284, RRID: AB_955387, Cambridge, UK) for 1h at room temperature. The membranes were developed with the ECL method according to the instructions (Millipore, USA) and detected on a Chemiluminescence imaging GeneGnome XRQ (Syngene, MD, USA). ImageJ software was used to calculate the relative density of proteins.

### Co-immunoprecipitation assay

Co-immunoprecipitation assay was performed as previous study [50]. After the CMs or heart tissue were transfected with AdV vectors, siRNAs or plasmids, the cells were washed with ice-cold lysis buffer containing a protease inhibitor mix (Sigma, Darmstadt, Germany). The lysates were incubated with a p21 antibody or a Sirt1 antibody for 1h and then with protein A/G-agarose beads (Millipore, USA) at 4°C for 12h. The immunoprecipitates were pelleted, washed and subjected to immunoblotting as described above using acetyl-lysine antibody (Cell Signaling Technology, #9441, RRID: AB_331805, MA, USA), ubiquitin antibody (Abcam, ab19247, RRID: AB_444805, Cambridge, UK), Sirt1, or p21 antibody.

### Mass spectrometry

To detect specific acetylated sites within the acetylated p21 peptide in CMs treated with or without Ad-Sirt1 or si-Sirt1, cell lysates were immunoprecipitated with an anti-p21 antibody and analyzed using SDS-PAGE. The gel was silver-stained to detect p21 and gel slices containing p21 bands were excised. Following reduction and alkylation, gel slices were digested with a modified sequencing grade trypsin (Sigma, Darmstadt, Germany). The digestion was carried out overnight at 37 °C and the fragmented peptides were extracted from the gel with 2% formic acid/67% acetonitrile. Then, the digestion mixture was packed with C18-resin (ThermoScientific, MA, USA). A capillary peptide trap was used to desalt and condense the tryptic peptides before separation on a reverse-phase 75 μm × 15 cm Chrom XP Eksigent column (AB SCIEX, MA, USA) packed with C18-resin. The column was directly mounted in the nanoelectrospray ion source (AB SCIEX, MA, USA) and the peptides were eluted by applying a linear gradient program (5–50% B 25 min, 50% B 30–35 min and 50–95% B 35–60 min, where mobile phase B was 0.1% formic acid, 95% HPLC grade acetonitrile, 5% HPLC grade water and mobile phase A was 0.1% formic acid, 5% HPLC grade acetonitrile, and 95% HPLC grade water) at a constant flow of 170 nl/ml. LC-MS/MS was carried out on a Triple TOF 5600 LC-MS (AB SCIEX, MA, USA).

### RNA isolation and real-time qPCR

Total RNA was extracted from isolated CMs or ventricular neonatal or adult mouse heart tissue samples using the E.Z.N.A. Total RNA Kit II (Norcross, GA, USA) according to the manufacturer’s instructions. The RNAs were treated with DNase I (Invitrogen, CA, USA) to exclude the possibility of DNA contamination. For the quantification of gene expression, cDNA was reverse transcribed from 1μg of total RNA according to the PrimeScript^TM^ RT Master Mix (TaKaRa, Dalian, China). Real time qPCR was performed with the SYBRs Premix Ex Taq^TM^ Kit (TaKaRa, Dalian, China) on Lightcycler480 (Roche, Basel, Switzerland). β-actin was used as a housekeeping control to normalize gene expression normalization using the ΔΔCt method. All primers were designed by Sangon Biotech (Shanghai, China) Co., Ltd. The sequences of the primers are as follows: Sirt1, 5′-TTGGCACCGATCCTCGAAC-3′ (forward), 5′-CCCAGCTCCAGTCAGAACTAT-3′ (reverse); Sirt2, 5′-AGCCAACCATCTGCCACTAC-3′ (forward), 5′-CCAGCCCATCGTGTATTCTT-3′ (reverse); Sirt3, 5′-CTACATGCACGGTCTGTCGAA-3′ (forward), 5′-GCCAAAGCGAAGTCAGCCATA-3′ (reverse); Sirt4, 5′-GGATGCATGCACAGAGTCCTG-3′ (forward), 5′-CTCAGTGAGGAACACGTCGC-3′ (reverse); Sirt5, 5′-TCTGGAAATCCACGGAACCT-3′ (forward), 5′-ACTGGGATTCTGGCGTCTTG-3′ (reverse); Sirt6, 5′-ATGTCGGTGAATTATGCAGCA-3′ (forward), 5′-GCTGGAGGACTGCCACATTA-3′ (reverse); Sirt7, 5′-GACTGAGCGTACTGCCCTTC-3′ (forward), 5′-ACAATGGTATCCCGAAGCTG-3′ (reverse); p21, 5′-CACAGCTCAGTGGACTGGAA-3′ (forward), 5′-CCACCACCACACACCATAGA-3′ (reverse); β-actin, 5′-TGCTGTCCCTGTATGCCTCTG-3′ (forward), 5′-TTGATGTCACGCACGATTTCC-3′ (reverse).

### MI

Healthy 6-week-old male C57BL/6J mice were purchased from the Laboratory Animal Center of Southern Medical University and singly housed in a temperature-controlled (22±1°C) environment with a 12 hour light/dark cycles with food and water available *ad libitum*. The animals were habituated to the animal facilities for at least 1 week before use. MI surgeries were performed as previous study [51]. Randomization was used to assign samples to the experimental groups and to collect and process data. The experiments were performed by investigators blinded to the treatment groups. MI was generated by ligating the LAD coronary artery in mice at 8-week-old (24-26g). For surgical MI, mice were anesthetized with 2% isofluorane. An ALC-V8S rodent ventilator (ALCBIO, Shanghai, China) was used to supply oxygen during the surgical procedure. Mouse chests were opened through the muscle between the fourth and the fifth intercostal space, and the pericardium was then removed. Then the LAD coronary artery was permanently ligated with a 5-0 silk suture (Ningbo Medical Needle Co., Ningbo, China). An analogous surgical operation was performed without occlusion of the coronary artery to generate sham-operated animals. After surgery, the thoracic muscle, thoracic wall and skin were closed with a 5-0 silk suture. Myocardial ischemia was confirmed by an ECG ST-segment elevation using an Animal Bio Amp (Adinstruments, NSW, Australia). After ligation, AAV9 vectors were immediately injected into the myocardium bordering the infarct zone using an insulin syringe with an incorporated 30-gauge needle. After surgery, the skin was disinfected and the animals were revived and maintained on a thermal insulation blanket. To prevent any postoperative discomfort, the animals received buprenorphine (0.03–0.06 mg/kg). EdU was administered intraperitoneally (500 μg per animal) on alternate days up to day 12 post-MI. Hearts were collected 28 days after infarction as described below.

### Echocardiography

To evaluate cardiac dimensions and function, transthoracic two-dimensional echocardiography was performed on mice anaesthetized with 2% isoflurane at 7, 14, 21, and 28 days after LAD ligation, using a Vevo 2100 Imaging System (Visual Sonics, ON, Canada) equipped with a 40-MHz probe. M-mode tracings in the parasternal short axis view were used to measure the left ventricular internal diameter at end-diastole (LVEDd) and end-systole (LVESd). These measurements were used to calculate the left ventricular fractional shortening (LVFS) and ejection fraction (LVEF).

### Tissue collection

Tissue collection was performed as previous study [2]. Mice were anaesthetized with 2% isoflurane and then sacrificed by injection of 10% KCl. The hearts and lungs were excised, briefly washed in 0.9% NaCl, weighed, and fixed in 10% formalin at room temperature. Then the hearts were embedded in paraffin and further processed for histology or immunofluorescence experiments.

### Masson’s trichrome staining

Paraffin-fixed heart tissue slides were de-paraffinized via xylene and re-hydrating through sequential incubations in ethanol (100%, 100%, 90%, 80%, and 70%) and water. Then, the serial slices were incubated in Weigert hematoxylin iron for 5 min, differentiated in HCl-ethanol and incubated in ponceau acid fuchsin for 5 min, phosphomolybdic acid for 5 min, and aniline blue (Leagene, Beijing, China) for 5 min. The fibrotic area was measured as the percentage of the total left ventricular area showing fibrosis and quantified with ImageJ software.

### Immunofluorescence analysis

For cells cultured *in vitro*, the culture medium was washed with PBS. Then, the cells were fixed with 4% ice-old paraformaldehyde (Leagene, Beijing, China). For the tissue slices, frozen sections (5μm) were fixed with acetone. Then, cells or slides were permeabilized with 0.2% Triton X-100 PBS and blocked with 1% BSA in PBS. When indicated, cells or slides immunostained with EdU were further processed using a Click-iT EdU Alexa Fluor 555 Imaging Kit (Invitrogen, CA, USA) to determine EdU incorporation according to the manufacturer’s instructions. Then, the cells or slides were incubated with primary antibodies, including the CM marker cardiac troponin T (Abcam, ab8295, RRID: AB_306445, Cambridge, UK), the cardiac fibroblast marker vimentin (Abcam, ab24525, RRID: AB_778824, Cambridge, UK), pH3 (Abcam, ab47297, RRID: AB_880448, Cambridge, UK), or Aurora B (Abcam, ab2254, RRID: AB_302923, Cambridge, UK), for 2h at room temperature, followed by incubation with goat anti-mouse IgG/Alexa Fluor 488 (Biosynthesis, bs-0296G-A488, RRID: AB_10893722, Beijing, China) or goat anti-rabbit IgG/Alexa Fluor 555 (Biosynthesis, bs-0295G-A555, RRID: AB_10893940, Beijing, China) or goat anti-chicken IgG/Alexa Fluor 647 (Abcam, bs-0310G-A647, RRID: AB_10892751, Cambridge, UK) secondary antibodies for 1h at room temperature. The cells or slides were subsequently washed and incubated with Hoechst 33342. CM numbers were counted using cTnT staining. CM borders were determined by staining of slides with WGA conjugated to Alexa Fluor 555 (Invitrogen, CA, USA) in PBS. CM apoptosis was defined by TUNEL staining (Roche, Basel, Switzerland) at 28 days after MI according to the manufacturer’s instructions. Image acquisition was performed with an LSM 880 confocal microscope (Zeiss, Oberkochen, Germany).

### Gene expression omnibus (GEO) data

The previously published microarray data that were reanalyzed here are available under the accession codes GSE50704, GSE51483, and GSE93269.

### Statistical analysis

All data are presented as the mean ± S.E.M. All statistical analyses were performed by SPSS 20.0 software (IBM, NY, USA) and only when a minimum of n = 5 independent samples was acquired. For statistical comparisons of two groups, an unpaired, two-tailed Student’s t-test was used; for comparisons of three or more groups, a one-way ANOVA followed by the least significant difference (LSD) post hoc test was used. We calculated the significance of each treatment as a *p* value, and *p*<0.05 was considered statistically significant.

## Supplementary Material

Supplementary Figures
