# Supplementary material for: Sirt1-inducible deacetylation of p21 promotes cardiomyocyte proliferation
Source: Aging (Albany NY). 2019 Dec 26;11(24):12546–67. doi: 10.18632/aging.102587 (PMC6949046; doi:10.18632/aging.102587)
Supplement: Supplementary Figures [file aging-11-102587-s001..pdf]

SUPPLEMENTARY FIGURES

A

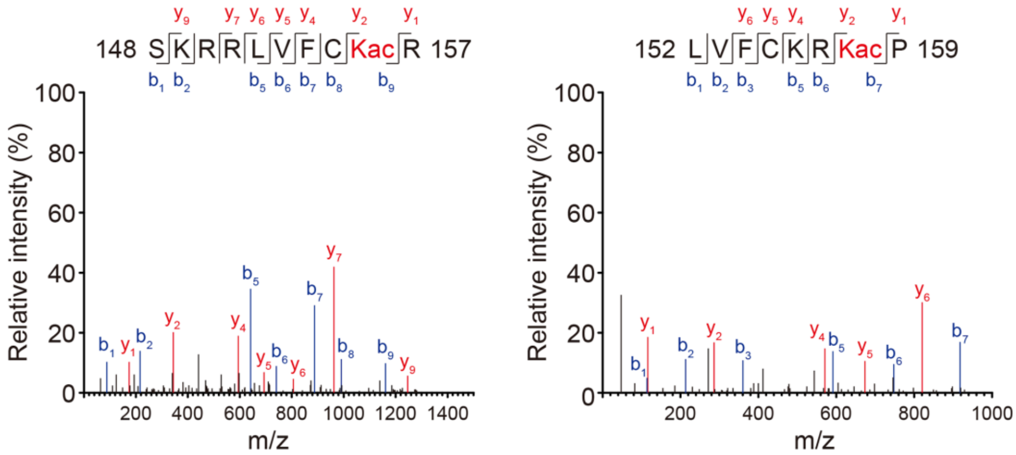

B

Mouse	FYHSKRRLVFCKRKP	159
Rat	FYHSKRRLVFCKRKP	164
Human	FYHSKRRLIFSKRKP	164
Chimpanzee	FYHSKRRLIFSKRKP	164
Dog	FYHSKRRLIFSKRKP	164
Cattle	FYHSKRRLICSKRKP	216
Pig	FYHSKRRLIFSKRKP	164

Supplementary Figure 1. Lysine K156 and K158 are the acetylation sites on p21. (**A**) NanoLC-MS/MS spectrum of acetylated p21 peptides containing lysines K156 and K158. (**B**) Sequence alignment of the region surrounding the K156 and K158 residues of p21.

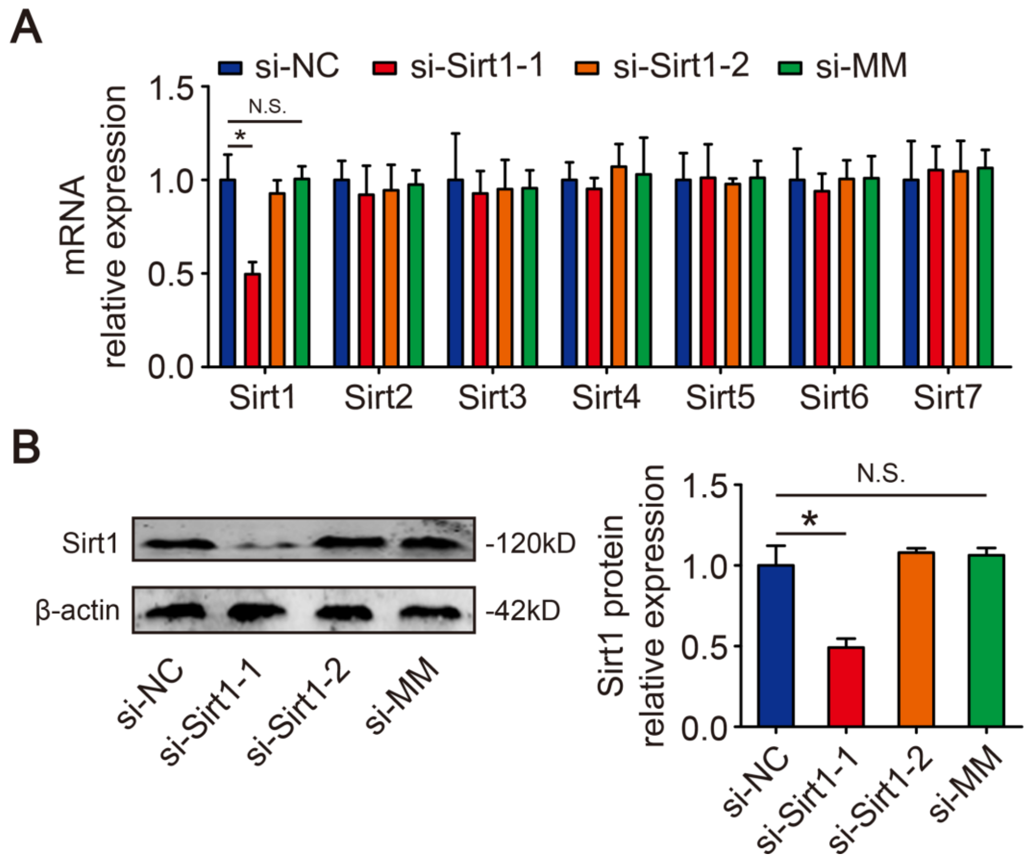

Supplementary Figure 2. Efficiency and specificity of Sirt1 knockdown in isolated CMs. (**A**) Isolated P1 CMs were transfected with si-NC, two Sirt1 siRNA, or mismatched control siRNA (si-MM), and the mRNA expression of Sirt1-7 were detected using RT-qPCR experiments (n=3). (**B**) Western blotting and quantitative analyses of Sirt1 protein levels in isolated P1 CMs transfected with si-NC, two Sirt1 siRNA, or si-MM. β-actin was used as a loading control (n=3). Statistical significance was calculated using a one-way ANOVA followed by the LSD post hoc test in A-B. **p*<0.05; N.S., no significance. data are presented as the mean ± S.E.M.

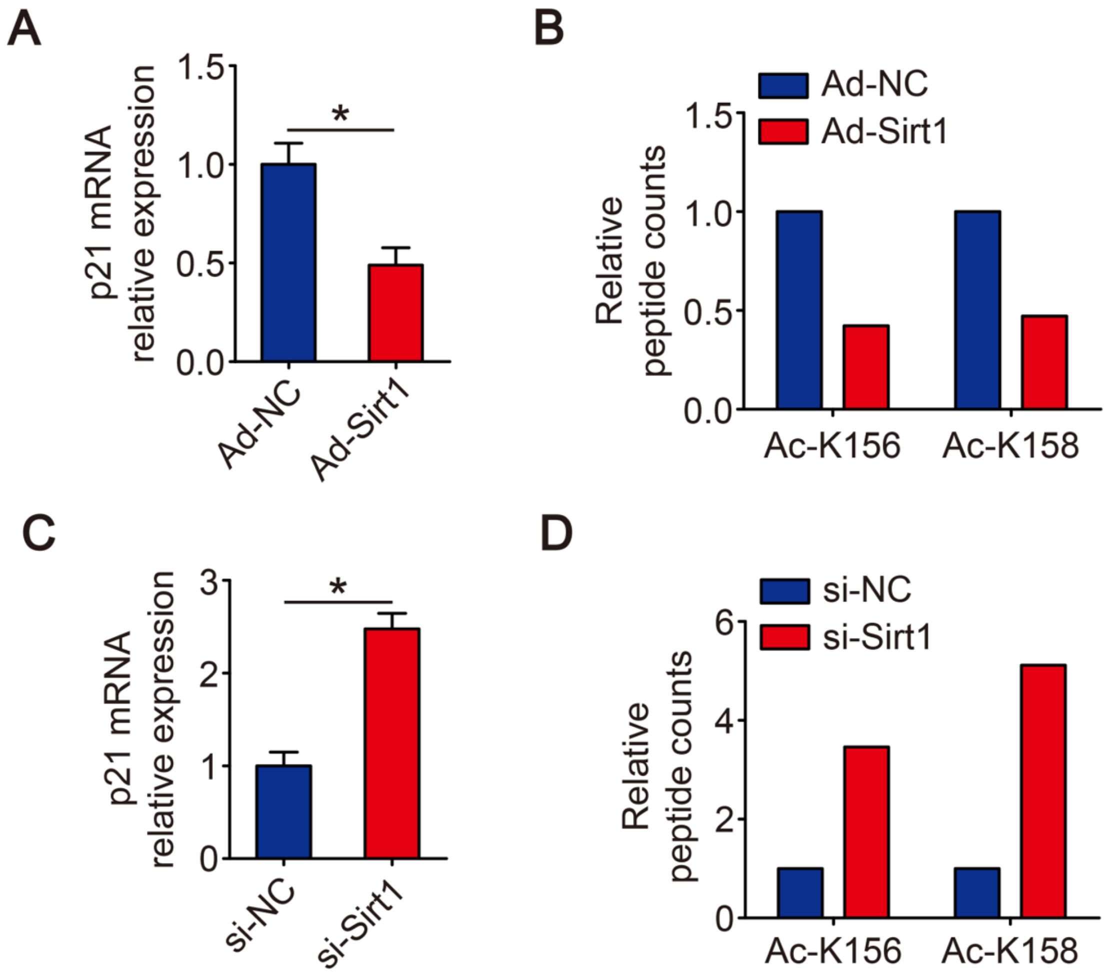

Supplementary Figure 3. Sirt1 decreases p21 mRNA expression and deacetylates p21 on K156 and K158. (**A**) Isolated P1 CMs were transfected with Ad-NC or Ad-Sirt1, and the mRNA expression of p21 was detected by RT-qPCR (n=3). (**B**) Relative acetylated K156 or K158 peptide counts in isolated CMs transfected with Ad-NC or Ad-Sirt1. (**C**) Isolated P1 CMs were transfected with si-NC or si-Sirt1, and the mRNA expression of p21 was detected by RT-qPCR (n=3). (**D**) Relative acetylated K156 or K158 peptide counts in isolated CMs transfected with si-NC or si-Sirt1. Statistical significance was calculated using a two-tailed unpaired Student's t-test in A and C. *$p<0.05$; data are presented as the mean ± S.E.M.

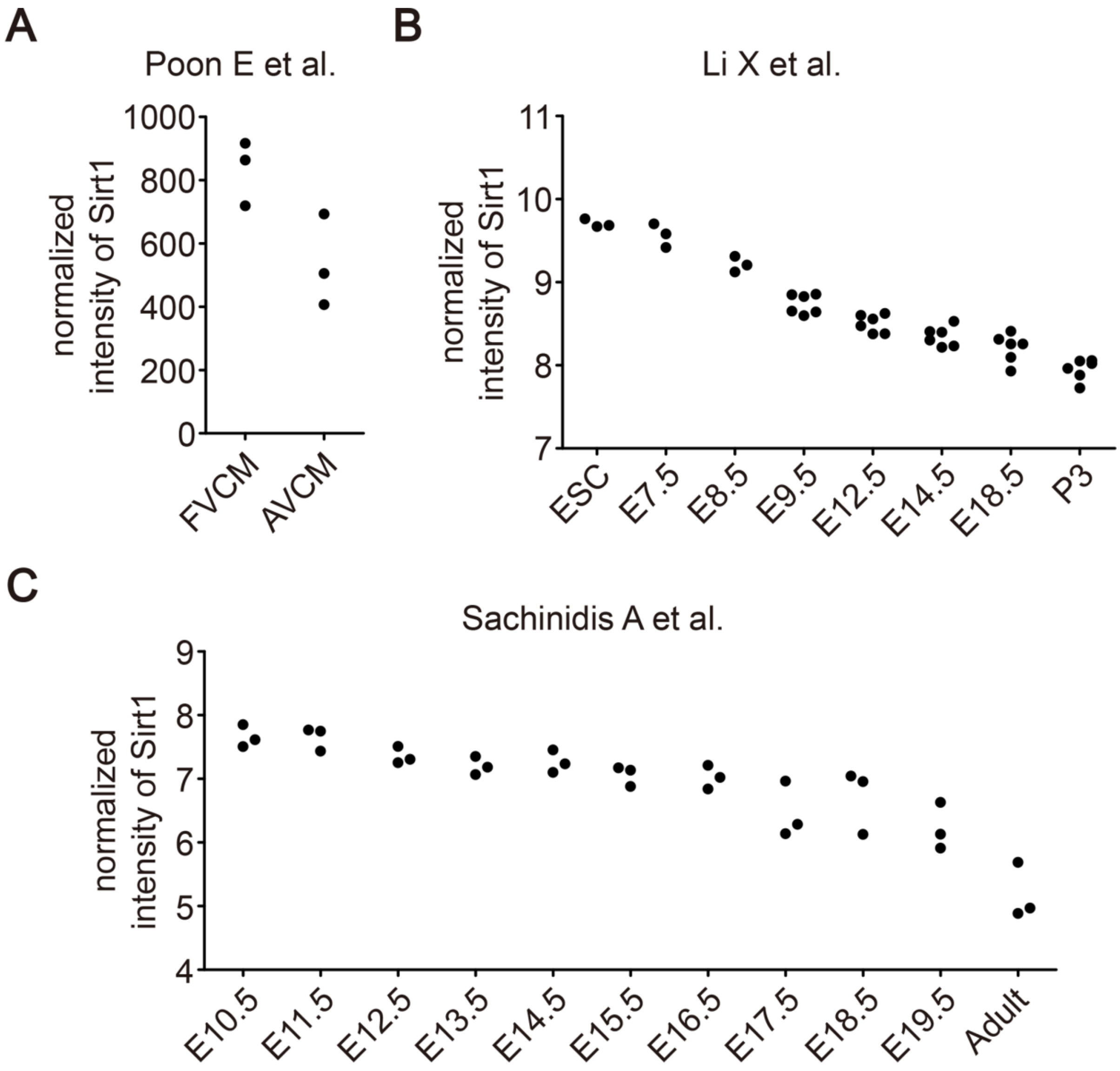

Supplementary Figure 4. Sirt1 is highly expressed in fetal hearts and a lowly expressed in adult hearts based on GEO data. (**A**) Normalized Sirt1 expression levels during human fetal ventricular cardiomyocytes (FVCM) and adult ventricular cardiomyocytes (AVCM) from GSE50704 data. (**B**) Normalized Sirt1 expression levels during mouse embryonic stem cells (ESCs), E7.5, E8.5, E9.5, E12.5, E18.5, and P3 hearts from GSE51483 data. (**C**) Normalized Sirt1 expression levels during mouse E10.5, E11.5, E12.5, E13.5, E14.5, E15.5, E16.5, E17.5, E18.5, and E19.5, and adult hearts from GSE93269 data.

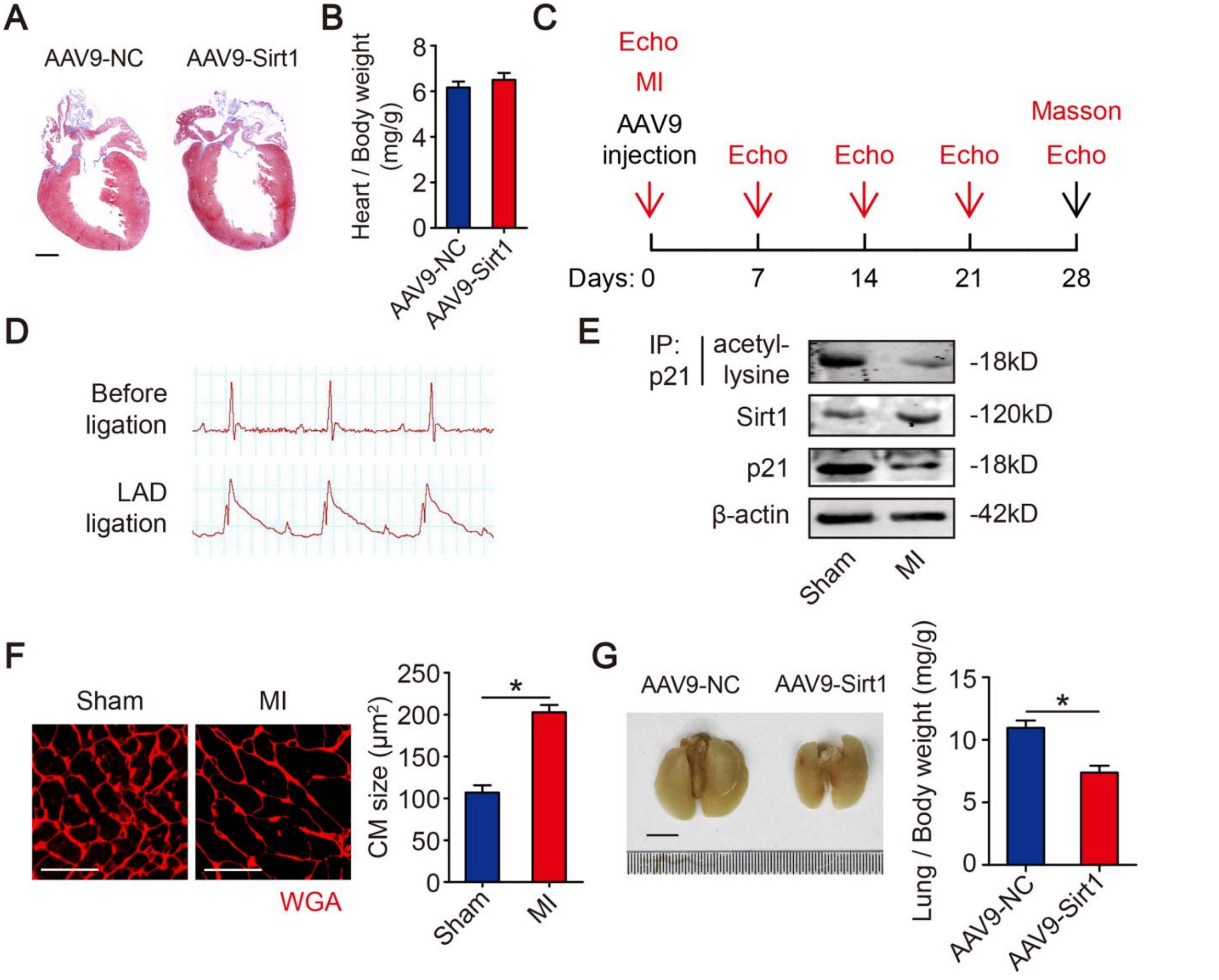

Supplementary Figure 5. Overexpression of Sirt1 does not change morphology in adult hearts and decreases lung weight in adult MI mice. (**A**) Masson staining of sagittal heart sections from the adult AAV9-NC and AAV9-Sirt1 groups. Scale bar, 1mm. (**B**) Ratios of heart weight-to-body weight in neonatal mouse hearts injected with AAV9-NC or AAV9-Sirt1 (n=5). (**C**) Schematic of the MI experiments in adult mice. Echo, echocardiography. (**D**) The adult mouse MI model was confirmed using an electrocardiogram ST-segment elevation. (**E**) Heart lysates were immunoprecipitated with a p21 antibody and analyzed by Western blotting using an acetyl-lysine antibody, and Western blotting was performed to evaluate Sirt1 and p21 protein expression for the Sham and MI groups; (**F**) WGA staining and quantitative analyses of CMs size in Sham and MI group. Scale bar, 50 μm. Quantitative analyses are representative of fields from 5 mice per group. (**G**) Ratios of lung weight-to-body weight in adult MI mouse hearts injected with AAV9-NC or AAV9-Sirt1 (n=5). Scale bar, 5mm. Statistical significance was calculated using a two-tailed unpaired Student's t-test in **B**, **F**–**G**. *p<0.05; data are presented as the mean ± S.E.M.
